# Two-stage multi-objective optimization for ICU bed allocation under multiple sources of uncertainty

**DOI:** 10.1038/s41598-023-45777-x

**Published:** 2023-11-02

**Authors:** Fang Wan, Julien Fondrevelle, Tao Wang, Antoine Duclos

**Affiliations:** 1 School of Computer Science, Univ Lyon, INSA Lyon, Univ Jean Monnet Saint-Etienne, Université Claude Bernard Lyon 1, Univ Lyon 2, DISP-UR4570, 69621 Villeurbanne, France; 2 Research On Healthcare Performance (RESHAPE), Université Claude Bernard Lyon 1, INSERM U1290, Lyon, France; 3 Health Data Department, Lyon University Hospital, Lyon, France

**Keywords:** Health services, Diseases, Health care

## Abstract

Due to the impact of COVID-19, a significant influx of emergency patients inundated the intensive care unit (ICU), and as a result, the treatment of elective patients was postponed or even cancelled. This paper studies ICU bed allocation for three categories of patients (emergency, elective, and current ICU patients). A two-stage model and an improved Non-dominated Sorting Genetic Algorithm II (NSGA-II) are used to obtain ICU bed allocation. In the first stage, bed allocation is examined under uncertainties regarding the number of emergency patients and their length of stay (LOS). In the second stage, in addition to including the emergency patients with uncertainties in the first stage, it also considers uncertainty in the LOS of elective and current ICU patients. The two-stage model aims to minimize the number of required ICU beds and maximize resource utilization while ensuring the admission of the maximum number of patients. To evaluate the effectiveness of the model and algorithm, the improved NSGA-II was compared with two other methods: multi-objective simulated annealing (MOSA) and multi-objective Tabu search (MOTS). Drawing on data from real cases at a hospital in Lyon, France, the NSGA-II, while catering to patient requirements, saves 9.8% and 5.1% of ICU beds compared to MOSA and MOTS. In five different scenarios, comparing these two algorithms, NSGA-II achieved average improvements of 0%, 49%, 11.4%, 9.5%, and 17.1% across the five objectives.

## Introduction

The Covid-19 pandemic has hit the whole world since the end of December 2019 on an unprecedented scale. Its unpredictability has major implications for stabilizing healthcare offers, leading the health authorities to call for a massive deprogramming of surgical activity in all healthcare facilities to increase care capacities, particularly in the ICU^1^. Many patients have seen their service postponed or cancelled without an expected resumption date. This measure could obscure the prognosis of patients, or even make them lose a potential chance^2,3^.

Therefore, optimizing the allocation of these resources (operating rooms, nurses, hospital beds, etc.) and improving the utilization of medical resources are important means to mitigate the potential gap between medical supply and demand to promote the upgrading of medical services. Table 1 presents a synthesis of previous works from the literature dealing with the allocation of medical resources.

**Table 1:** Literature review related to the allocation of medical resources.

Ref.	Backgrounds	Uncertain factors	Objectives	Method
Zhang et al.^17^	Solve the problem of bed arrangement in eye hospital	Patient number, LOS	Waiting time	Markov process, linear programming
Liu et al.^15^	Determine the surgery sequence and doctors for elective patients	Surgery duration	Operating time, resource utilization	Genetic algorithm
Peng et al.^14^	Allocate operating rooms, surgery dates and ICU beds for elective patients	LOS, surgery duration, patient number	Resource utilization, costs	Robust optimization, column generation
Shen and Deng^18^	Arrange 5 types of ophthalmic patients to be hospitalized	–	Bed turnover, patient satisfaction	Particle swarm optimization
Davis and Fard^12^	Determine bed occupancy for 4 types of patient beds in COVID-19 situation	Patient number, LOS	Bed utilization	Statistical model
Tsai et al.^16^	Determine surgery start time and sequence for elective and emergency patients	Patient number, surgery duration,	Overtime cost	Simulation optimization, Laplace transform
Bekker et al.^11^	Predict the number of occupied ICU and clinical beds due to COVID-19	Patient number, LOS	Tardiness costs	Statistical model, queuing model
Wang et al.^19^	Arrange surgery and ICU beds for elective patients	LOS, surgery duration	Bed utilization	Fuzzy model, Genetic Algorithm
Li et al.^8^	Schedule the operation rooms for emergency and elective surgeries	Surgery duration	Surgery profit	Grey wolf algorithm, robust optimization
Maghzi et al.^20^	Allocate operating rooms and medical equipment to specific patients	Patient preparation, surgery duration	Cost, surgery number	Fuzzy model, NSGA-II
Fattahi et al.^21^	Allocate equipment resources and service resources to patients during COVID-19	Patient number	Patient admission, resource utilization	Stochastic programming
Chang et al.^22^	Design casualty collection points and allocate medical resources for earthquakes	Arrivals time; service times	Delivery time, triage waiting time	Rapid-screening and particle algorithm
Wang et al.^23^	Research operating room scheduling under non-operating room anesthesia mechanism	Emergency patient arriving	OR utilization, cost	Heuristic algorithm

The healthcare of surgical patients usually consists of operative and postoperative care. The operative uses the corresponding upstream resources and the postoperative uses the corresponding downstream resources^4^. Given that most upstream resources are relatively expensive and scarce, especially for the use of operating rooms (ORs), the mainstream of extant research focuses on the planning issues of upstream resources, while simply assuming ample sufficiency of downstream resources (e.g., inpatient beds)^5,6^. However, a shortage of downstream resources, such as inpatient or ICU beds, not only hinders the timely treatment of patients but also adversely impacts the utilization of related resources in the operating rooms. Ordu et al.^7^ introduced a hybrid forecasting-simulation–optimization model, using statistical datasets from A&E, outpatient, and inpatient services, to predict patient demand for physicians, nurses, and beds, thereby facilitating better planning for both upstream and downstream resources.

Additionally, in real healthcare systems, numerous inevitable uncertainties complicate resource allocation and staff scheduling, e.g., the number of emergency patients, surgery durations, and inpatient length-of-stay (LOS)^8^. Problems stemming from uncertain demand are typically addressed using stochastic programming or robust optimization^9,10^. These methods often fundamentally assume that variables adhere to a known probability distribution^11,12^. However, the underlying distribution is frequently unknown.

Furthermore, it's often essential to consider the benefits for both hospitals and patients. Akin and Ordu^13^ devised a novel patient-centered two-stage optimization approach to determine the required number of nurses and to schedule their shifts. To cater to the interests of multiple stakeholders, a multi-objective problem typically arises. For instance, potential objective functions might include minimizing patient waiting time, maximizing resource utilization, and maximizing the emergency reception rate^14–16^.

Considering the multiple uncertainties in ICU bed allocation and aiming to simultaneously reduce patient waiting time, increase patient admission rate, and improve bed occupancy rate, this study introduces a two-stage bed allocation model for three categories of patients. This model holistically balances these objectives. Among these categories: An “elective patient” is someone scheduled for surgery or treatment in advance and is not in an emergency situation. An “emergency patient” requires immediate medical care due to a sudden and critical condition. A “current ICU patient” refers to someone already receiving treatment in the ICU^24,25^. Given the proven efficacy of the genetic algorithm in solving medical resource allocation problems^26–28^, this paper employs the improved NSGA-II to address the issue.

This paper offers three main contributions:

First, it studies the ICU bed allocation problem for three categories of patients, taking into account various uncertainty factors associated with these groups.

Second, a two-stage multi-objective model is proposed with the objectives of minimizing the number of required ICU beds, maximizing resource utilization, and admitting the maximum number of patients.

Lastly, to evaluate the effectiveness of the model and algorithm, a variety of scenarios are presented, and comparisons are made with other methods.

The remainder of this paper is structured as follows. "The two-stage model formulation" section shows the problem description and formulation. In "Solution approach for the two-stage model" section, improved NSGA-II and solution approach are introduced. "Case study" section studies a real hospital case, and "Comparison experiments" section presents the numerical results, proving the superiority of the model and algorithm. “Conclusion” section concludes the paper and outlines future research directions.

## The two-stage model formulation

The problem of ICU bed allocation is a complex issue that involves multiple sources of uncertainty. To effectively address this complexity, the problem has been decomposed into two different stages. In the first stage, ICU beds are allocated to emergency patients, elective patients, and current ICU patients, given the uncertainties regarding the number and LOS of emergency patients. The second stage based on the results of the first phase, addressing uncertainties not only related to emergency patients but also incorporating uncertainties associated with elective patients and current patients.

### Allocation model of the first stage

In the first stage, there are three primary optimization objectives. The first aims to maximize the bed occupancy rate, the second seeks to minimize the delay index for elective patients, and the third emphasizes maximizing the admission rate for emergency patients. The parameters, variables, and mathematical formulas for the first stage are described below:

**Indices and sets:**

$T$: Set of planning horizon indexed by $t$,

$el$: Set of elective patients indexed by $i$,

$N$: Set of current ICU patients indexed by $n$,

**Parameter:**

$Q$: Number of available ICU beds in hospital,

$Exp_{i}$: Expected admission date of elective patient *i*,

$LOSel_{i}$: LOS of elective patient *i*,

$Loss_{i}$: Loss of chance if postponed for elective patient *i*,

$Rem_{n}$: Remaining LOS of current ICU patient *n*,

$Mp_{t}$: Maximum number of emergency patients on the day $t$,

$\lambda _{t}$: Parameter of Poisson distribution on the day $t$,

**Variables:**

${\text{e}}m_{t}$: Number of emergency patients arriving on the day $t$, indexed by $j$.

$Cu_{t}$: All ICU inpatients on the day $t$,

$LOSem_{jt}$: LOS of the emergency patient $j$ on the day $t$,

**Decision variables:**

$$X_{it} = \left\{ \begin{array}{ll} {1,} & \quad Elective \, patient \, i \, is \, admitted \, o n \, the \, day \, t \hfill \\ {0,} & \quad otherwise \end{array} \right.$$

**Mathematical model:**

1 $$\max f_{11} = \left(\sum\limits_{t = 1}^{T} \frac{Oc_{t}}{Q} \right)/T$$

2 $$\min f_{12} = \sum\limits_{t = 1}^{T} {\sum\limits_{i = 1}^{el} {\left( {Loss_{i} \times (t - Exp_{i}) \times x_{it}} \right)} } /\sum\limits_{t = 1}^{T} {\sum\limits_{i = 1}^{el} {x_{it}} }$$

3 $$\max f_{13} = \sum\limits_{t = 1}^{T} {\frac{em_{t}}{{Mp_{t}}}}$$

4 $$Cu_{t} = \sum\limits_{i = 1}^{el} {\sum\limits_{k = t - LOSel_{i} + 1}^{t} {x_{ik}} } + \sum\limits_{k = t - LOSem_{jk} + 1}^{t} {\sum\limits_{j = 1}^{em_{k}} {em_{jk}} } + \sum\limits_{Rem_{n} \ge t} {cu_{n}} ,\quad \forall \, t$$

5 $$O_{{\text{c}}t} = \sum\limits_{i = 1}^{el} {x_{it}} + em_{t} + Cu_{t}, \quad \forall \, t$$

6 $$\sum\limits_{i = 1}^{el} {x_{it}} + Cu_{t} \le Q{, } \quad \forall \, t$$

7 $$\sum\limits_{t = 1}^{T} {x_{it}} \le 1{, } \quad \forall \, t$$

8 $$em_{t}\sim P(\lambda_{t}),\quad\forall \, t$$

9 $$em_{t} \le Mp_{t} = \max P(\lambda_{t}), \quad \forall \, t$$

10 $${\text{e}}m_{t} \in N, \quad \forall \, t$$

11 $$Cu_{t} \in N, \quad \forall \, t$$

12 $$LOSem_{jt} \in N, \quad \forall \, j{,} \;\;\forall \, t$$

Equations (1–3) define the three optimization objectives of the first stage. Equation (4) represents the patients who are still occupying the beds on the day $t$. Equation (5) calculates the total number of patients in the ICU on the day $t$. Equation (6) indicates that the number of elective patients and current ICU patients does not exceed $Q$. Because the number of emergency patients is uncertain, the total number of three types of patients may exceed $Q$. Equation (7) indicates that elective patients will only be served once. Equation (8) supposes that emergency patients follow Poisson distribution^29,30^. Equation (9) indicates that the number of emergency patients admitted does not exceed the maximum number in the Poisson distribution. Equations (10–12) represent the range of variables, the number of emergency patients, the number of inpatients and the LOS of patients are integers.

### Allocation model of the second stage

In the second stage, further sources of uncertainty, particularly the LOS for elective and current ICU patients, are taken into account. This variation in LOS can arise as some patients might necessitate a prolonged stay due to disease progression or clinical complications. This stage emphasizes two objectives. Firstly, it seeks to minimize deviations from the initial bed arrangement established in the first stage, ensuring stability and consistency in ICU bed allocation. Secondly, the aim is to reduce the number of additional beds needed while guaranteeing accommodation for all patients. The parameters, variables, and mathematical formulas for this stage are detailed below:

**Variables:**

$R_{i}$: The random number that the elective patient $i$ extends LOS,

$R_{n}$: The random number that the current ICU patient $n$ extends LOS,

${\text{e}}m_{t}$: Number of emergency patients arriving on day $t$, indexed by $j$,

$Cu_{t}$: All ICU inpatients on the day $t$,

$LOSem_{jt}$: LOS of the emergency patient $j$ on the day $t$,

$NLOSel_{i}$: LOS of elective patient $i$ in the second stage,

$NRem_{n}$: Remaining LOS of current ICU patient $n$ in the second stage,

$S_{1}{{ = \{ }}Bedarr_{11},...,Bedarr_{1T}\}$: Bed allocation for three categories of patients during $T$ Days in the first stage,

$Q_{1}{{ = \{ }}q_{11},...,q_{1T}\}$: The required number of ICU beds every day to meet the needs of three categories of patients in the first stage,

$S_{2}{{ = \{ }}Bedarr_{21},...,Bedarr_{2T}\}$: Bed allocation for three categories of patients during $T$ Days in the second stage,

$Q_{2}{{ = \{ }}q_{21},...,q_{2T}\}$: The required number of ICU beds every day to meet the needs of three categories of patients in the first stage.

**Mathematical model:**

13 $$\max f_{21} = \sum\limits_{t = 1}^{T} {{(}Bedarr_{1t} \cap Bedarr_{2t}{)}}$$

14 $$\min f_{22} = \max (q_{21} - q_{11},...,q_{2T} - q_{1T})$$

15 $$\left( {\sum\limits_{t = 1}^{T} \frac{Oc_{t}}{Q} } \right)/T \ge f_{11}$$

16 $$\sum\limits_{t = 1}^{T} {\sum\limits_{i = 1}^{el} {\left( {Loss_{i} \times (t - Exp_{i}) \times x_{it}} \right)} } /\sum\limits_{t = 1}^{T} {\sum\limits_{i = 1}^{el} {x_{it}} } \le f_{12}$$

17 $$\sum\limits_{t = 1}^{T} {\frac{em_{t}}{{Mp_{t}}} \ge f_{13}}$$

18 $$Cu_{t} = \sum\limits_{i = 1}^{el} {\sum\limits_{k = t - NLOSel_{i} + 1}^{t} {x_{ik}} } + \sum\limits_{k = t - LOSem_{jk} + 1}^{t} {\sum\limits_{j = 1}^{em_{k}} {em_{jk}} } + \sum\limits_{NRem_{n} \ge t} {cu_{n}} ,\quad \forall \, t$$

19 $$O{\text{c}}t = \sum\limits_{i = 1}^{el} {x_{it}} + em_{t} + Cu_{t}{, } \quad \forall \, t$$

20 $$\sum\limits_{i = 1}^{el} {x_{it}} + Cu_{t} \le Q, \quad \forall \, t$$

21 $$\sum\limits_{t = 1}^{T} {x_{it}} \le 1, \quad \forall \, i$$

22 $$em_{t}\sim P(\lambda_{t}), \quad \forall \, t$$

23 $$em_{t} \le Mp_{t} = \max P(\lambda_{t}), \quad \forall \, t$$

24 $$LOSel_{i} \times (1 + R_{i}) \le NLOSel_{i},\quad \forall i$$

25 $$NLOSel_{i} \le LOSel_{i} \times (1 + R_{i}) + 1, \quad \forall i$$

26 $$Rem_{n} \times \left( {1 + R_{n}} \right) \le NRem_{n}, \quad \forall n$$

27 $$NRem_{n} \le Rem_{n} \times \left( {1 + R_{n}} \right) + 1, \quad \forall n$$

28 $${\text{e}}m_{t} \in N, \quad \forall \, t$$

29 $$Cu_{t} \in N, \quad \forall \, t$$

30 $$R_{i}, \, R_{n} \in Q, \quad \forall i, \;\; \forall n$$

31 $$LOSem_{jt} \in N, \quad \forall j,\;\;\forall t$$

32 $$NLOSel_{i} \in N, \quad \forall i$$

33 $$NRem_{n} \in N, \quad \forall n$$

Equations (13–14) delineate the two optimization objectives for the second stage. Specifically, Eq. (13) maximizes the congruence between the daily bed arrangements across two stages. A high degree of similarity implies that even with changes in a patient's LOS, the bed arrangement remains largely consistent, bolstering the stability of hospital scheduling. Equation (14) aims to minimize the need for additional ICU beds when patients extend their LOS. Equations (15–17) ensure that the new solution maintains the objectives set in the first stage. Equations (18–23) echo the constraints from the first stage. Equations (24–27) indicate scenarios where patients in the second stage extend their LOS, with the resultant extended LOS being rounded up to the nearest integer. Lastly, Eqs. (28–33) represent the range of variables, the number of patients and the extended LOS of patients are integers.

## Solution approach for the two-stage model

### Data features of patients

The data features of the three types of patients are different. The following is an introduction to the features of these patients.

#### The features of emergency patients

**Figure 1:**
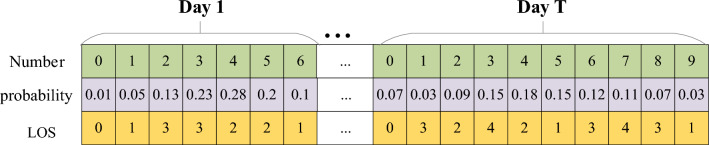
Data features of emergency patients.

The number of emergency patients per day is uncertain. The Poisson distribution, which describes the number of random events in a given unit of time (or space) as detailed by Nygren et al.^31^, can be utilized to represent this variability. Accordingly, the Poisson distribution is employed to formulate the probability distribution of emergency patient arrivals, as denoted in Eq. (34), where $P(X = k)$ represents the probability when the number of emergency patients is $k$, and $\lambda$ is the parameter of the Poisson distribution. The data features of emergency patients include the number of emergency patients and corresponding probability and random LOS, as shown in Fig. 1. For example, on day 1, the probability of 1 emergency patient is 5%, and the LOS is 1. The probability of 2 emergency patients is 13%, and their LOS is 1 and 3, respectively.

34 $$P(X = k) = \frac{{\lambda^{k} }}{k!}e^{ - \lambda } ,k = 0,1,2...$$

#### The features of elective patients

The data features of elective patients include the ID number, expected admission date, LOS, and “Loss of Chance if Postponed”. The latter refers to the potential detriment due to the real admission date being later than initially expected. A few literatures address this parameter^32,33^. In our research, the “Loss of Chance if Postponed” is categorized as mild (0.1), moderate (0.5), or severe (0.9). A value of 0.9 suggests that a delay in the patient’s admission would result in significant repercussions, thus warranting prioritization of the patient’s request. Conversely, a value of 0.1 suggests that postponement might be reasonable in the face of ICU bed shortages.

**Figure 2:**
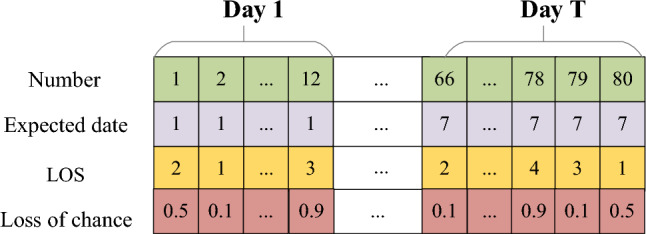
Data features of elective patients.

“Delay index” is used to measure the total loss caused by delay, which is equal to “delay days” × “Loss of chance if postponed”. As shown in Fig. 2, elective patient 1 expects to be admitted to ICU on day 1, but his admission is accepted on day 3, so his delay index is (3–1) × 0.5.

#### The features of current ICU patients

**Figure 3:**
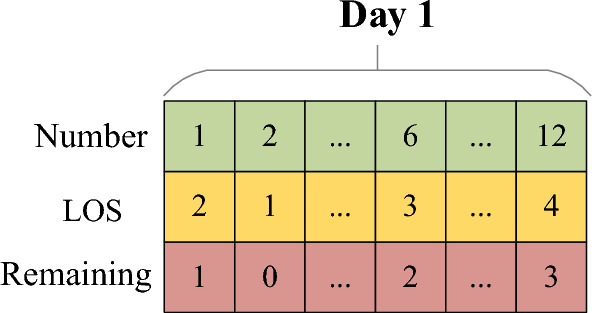
Data features of current ICU patients.

The data features of current ICU patients include the ID number of patients, LOS and remaining LOS, as shown in Fig. 3, for example, the LOS of current ICU patient 1 is 2, and his remaining LOS on day 1 is 1.

### The process of NSGA-II

**Figure 4:**
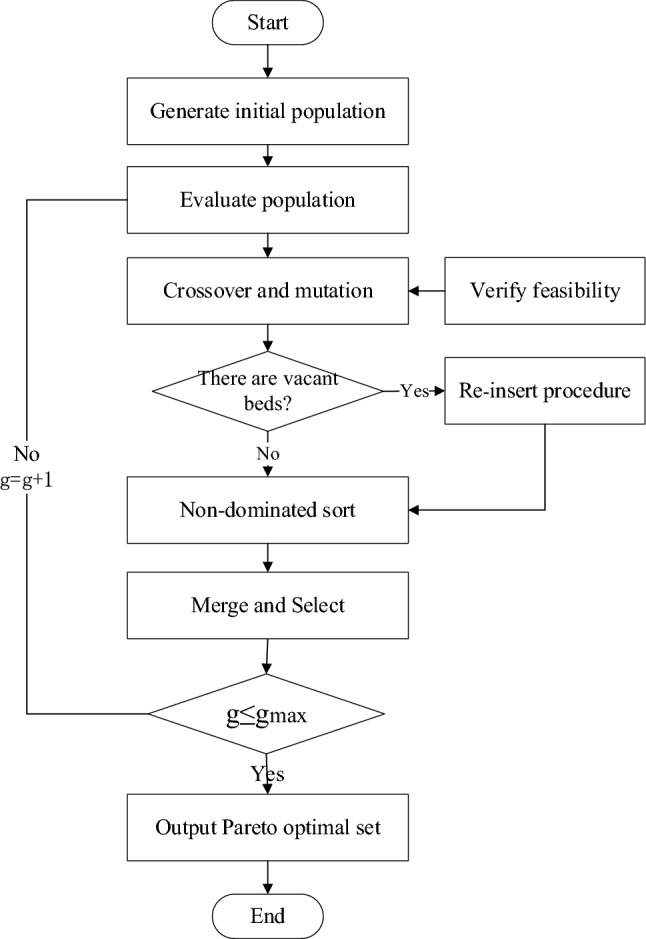
The framework of NSGA-II.

This paper mainly uses NSGA-II to solve this multi-objective problem with uncertainty. The framework of NSGA-II is shown in Fig. 4. The main steps are described in the following subsections.

#### The individual generation

**Figure 5:**
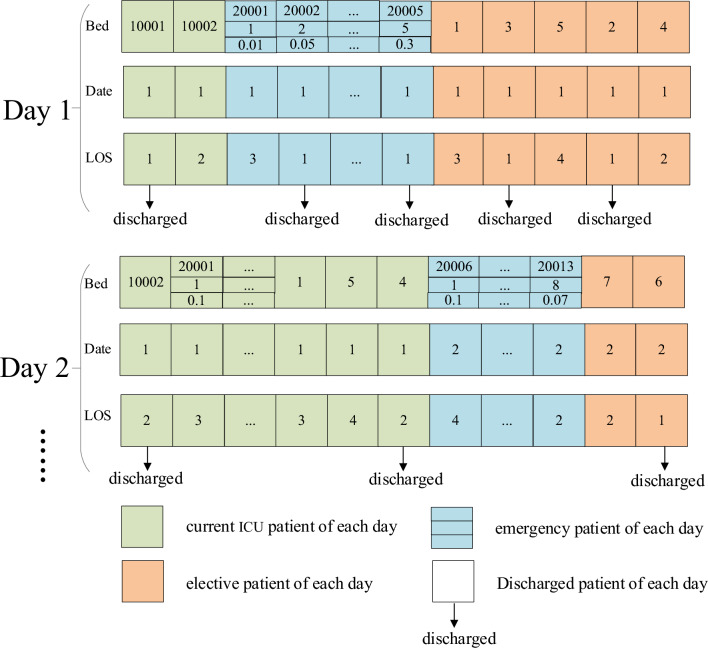
The coding method of the individuals.

An individual consists of bed assignments for all days, and the bed assignments for each day are generated by three steps: (1) Generate bed occupation of current ICU patients; (2) Generate uncertain numbers of emergency patients; (3) Select elective patients. The combined total of patients should not exceed the available number of beds. For clarity, different coding prefixes are used: current ICU patients have a prefix of 10,000−, emergency patients have 20,000−, and elective patients have 1−. This coding system is illustrated in Fig. 5

#### Initial population generation

**Figure 6:**
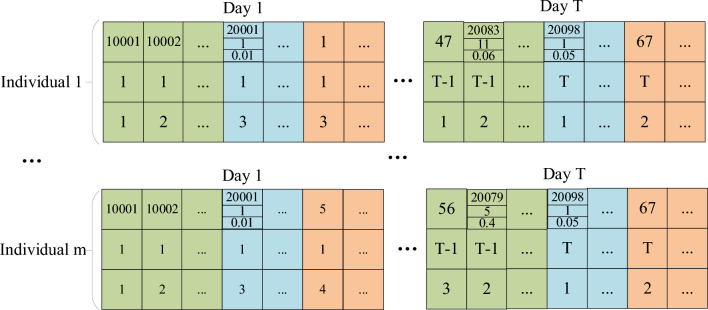
Generation of the initial population.

The initial population consists of a set of individuals, as shown in Fig. 6.

#### Crossover and mutation

Given that bed assignments for current ICU and emergency patients must be prioritized, crossover and mutation operations are only applied to elective patients. Due to the varying LOS for elective patients, bed constraints during their LOS must be observed during gene exchange operations.

**Figure 7:**
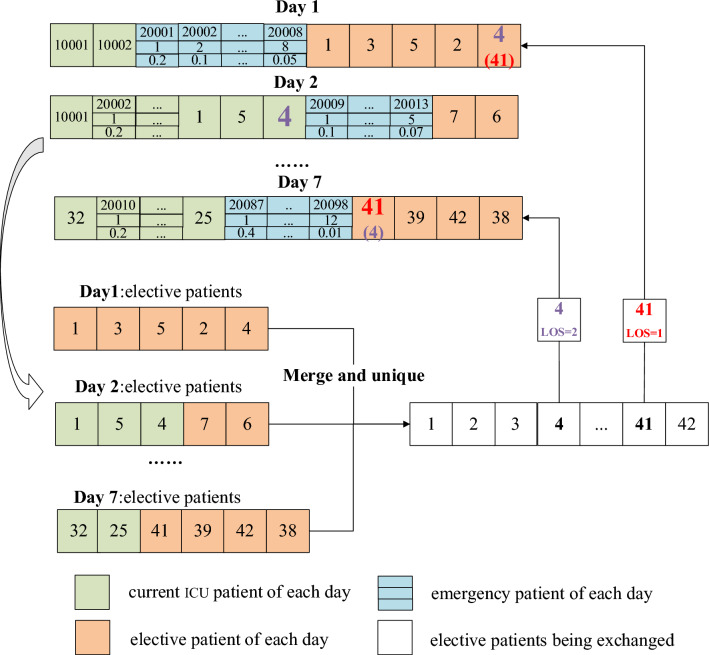
Crossover operation of individuals.

**Figure 8:**
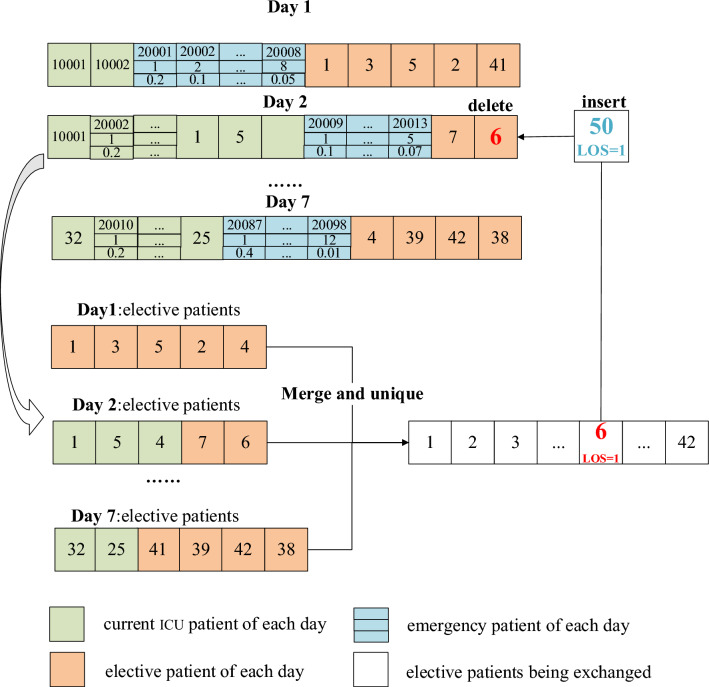
Mutation process of individuals.

For the crossover operation, two elective patients are randomly selected and their admission dates are exchanged. This operation ensures adherence to bed constraints and considers the expected admission date, as illustrated in Fig. 7. For the mutation operation, an elective patient is randomly chosen, their admission record is deleted, and then another patient, whose LOS is not longer than the original patient's, is inserted. This process is depicted in Fig. 8.

#### Re-insert procedure

**Figure 9:**
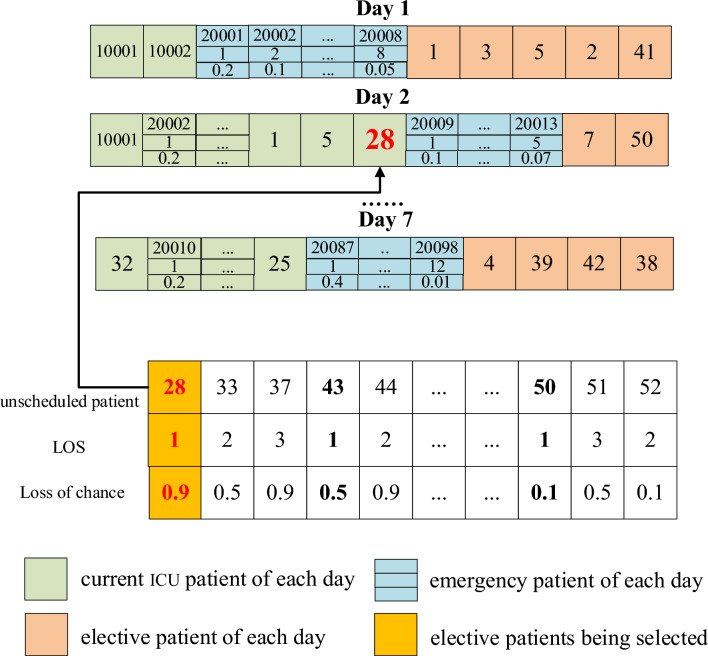
Re-insert process.

Due to variations in patients’ LOS, there may be vacant ICU beds following the crossover and mutation operations. To enhance bed utilization, patients with shorter LOS and a larger loss of chance are reintroduced. This procedure is termed the “Re-Insertion Process”, depicted in Fig. 9.

### NSGA-II for the first stage

The procedure of NSGA-II solving the first stage model is shown in Algorithm 1.

**Algorithm 1:**
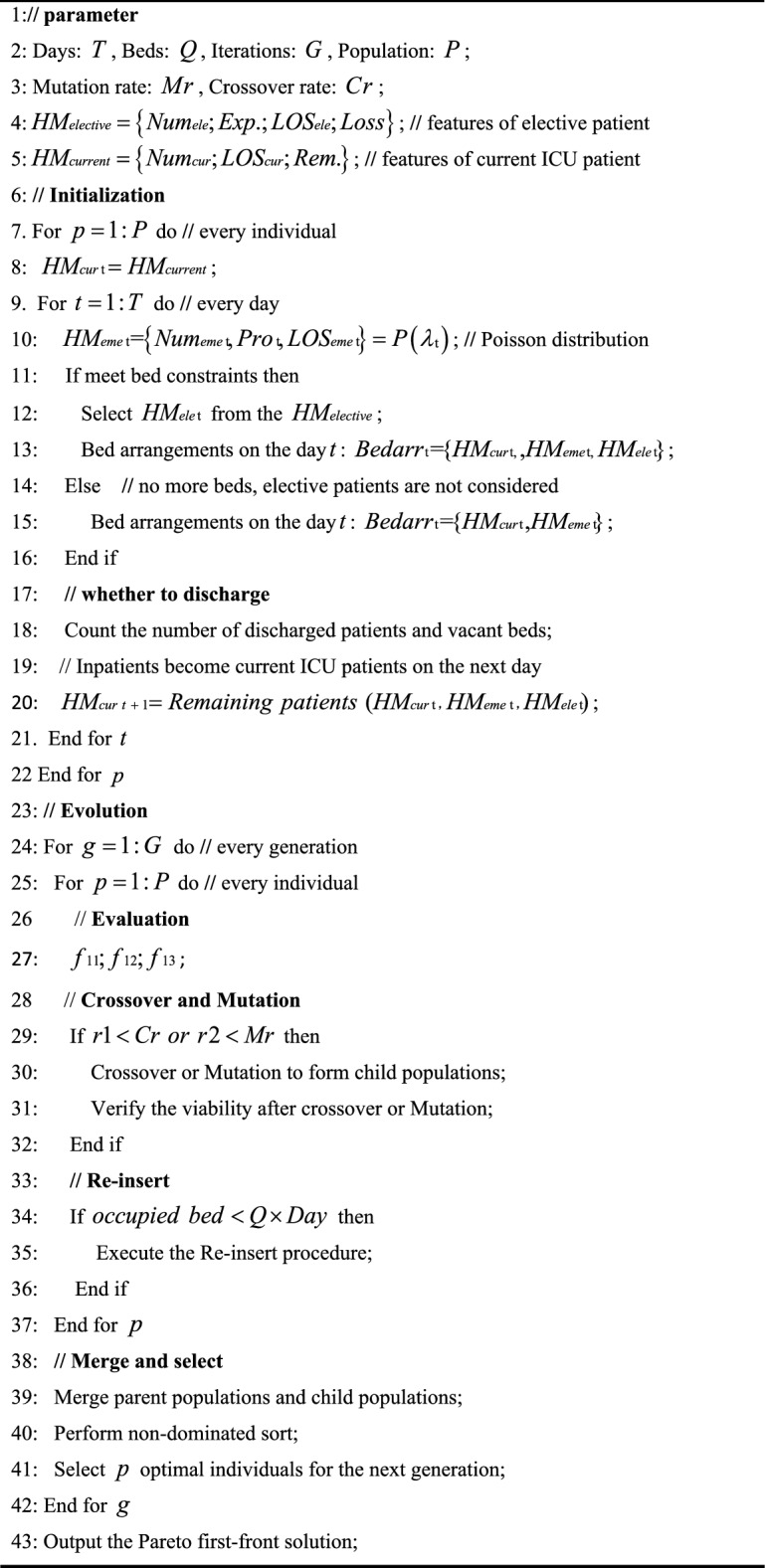
NSGA-II for the first stage.

### NSGA-II for the second stage

When the LOS for certain patients is extended, limited ICU beds might result in some patients not being admitted on the day originally scheduled in the first stage. Consequently, an extended LOS requires validation to ensure that the optimal solutions from the first stage still meet bed constraints. If affirmed, the algorithm outputs the optimal solutions. If not, three alternative strategies are employed to achieve a new optimal solution. The procedure of NSGA-II for solving the second stage model is detailed in Algorithm 2.Algorithm 2: NSGA-II for the second stage

**Algorithm 2:**
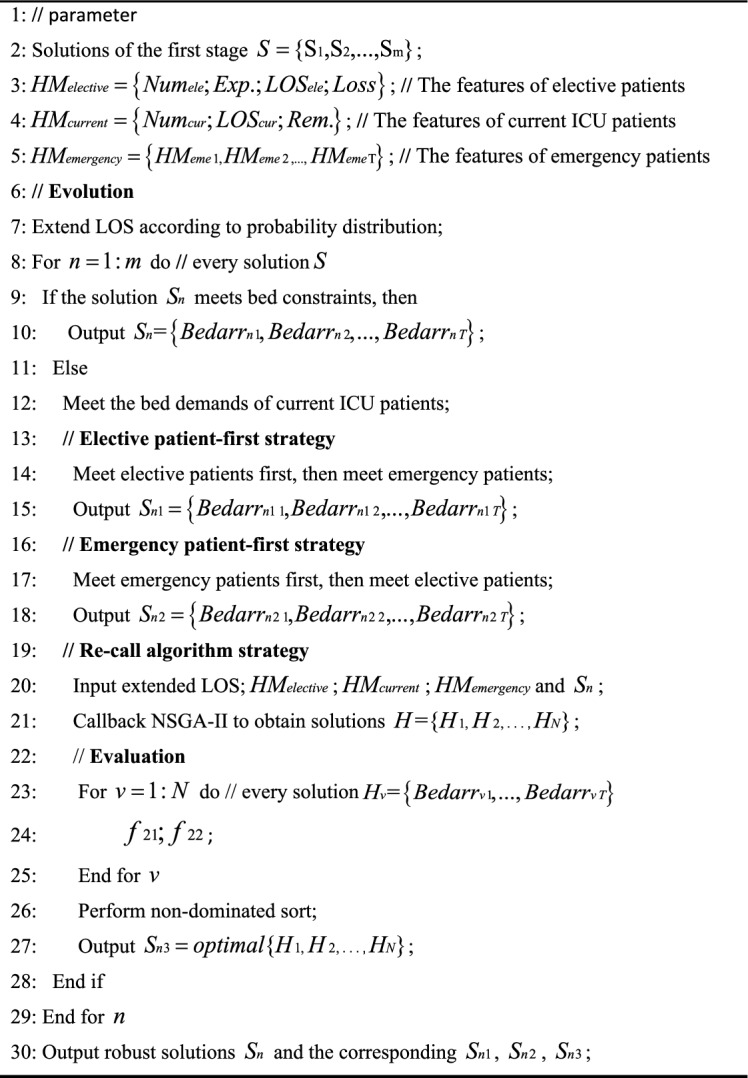
NSGA-II for the second stage.

## Case study

To evaluate the performance of the models and algorithms, we examined real-world cases from a hospital in Lyon, France. This hospital has a total of 24 ICU beds, with a planning horizon spanning one week. We simplified and privately treated the raw data. During the period, there were up to 80 elective patients and 3 current ICU patients. The daily number of emergency patients follows a Poisson distribution^34^. The parameter $\lambda$, according to the property of Poisson distribution, can be estimated by the average number of emergency patients over a period. These data are presented in Table 2. Additionally, the parameters for NSGA-II, derived from experimental tests, are detailed in Table 3.

**Table 2:** The data features of three types of patients.

Date	Emergency patients				Elective patients				Current ICU patients		
	Code	Num.	Pro.	LOS	Code	Exp.	LOS	Loss	Code	LOS	Rem.
Day1	–	0	0.01	0	1	1	3	0.1	10001	3	1
	20001	1	0.05	1	2	1	2	0.1	10002	4	2
	20002	2	0.13	3	3	1	1	0.5	10003	2	1
	…	…	…	…	…	…	…	…			
	20013	12	0.03	4	…	…	…	…			
	…	…	…	…	16	1	3	0.9			
…	…	…	…	…	…	…	…	…	…	…	…
Day7	–	0	0.07	0	73	7	1	0.9	……		
	20062	1	0.03	2	74	7	3	0.5			
	20063	2	0.09	4	75	7	2	0.5			
	…	…	…	…	…	…	…	…			
	20070	15	0.03	3	…	…	…	…			
	…	…	…	…	80	7	1	0.1

**Table 3:** The settings of algorithm parameters.

Parameter	Value	Parameter description
P	100	Number of individuals
G	200	Number of iterations
Mr	0.1	Mutation rate
Cr	0.7	Crossover rate
Q	24	Available ICU beds
T	7	Planning horizon
el	100	Number of elective patients
N	3	Number of current patients

### Result analysis of the first stage

In the first stage, the bed requirements for current ICU patients are addressed initially, followed by emergency patients, and finally elective patients. Table 4 presents the first-front solutions ordered by Pareto optimality. Columns 1, 2, and 3 display the numbers of the three types of patients admitted in these solutions. The 4th column represents the first objective: the bed occupancy rate. The 5th column details the second objective: the delay index. The 6th column highlights the third objective: the emergency admission rate. For instance, if the Poisson distribution predicts a maximum of 12 emergency patients on day 1, and only 3 emergency patients arrive, the emergency admission rate for that day stands at 3/12, or 25%. The three objectives are then averaged over the planning horizon.

**Table 4:** The Pareto first-front solutions of the first stage.

Solution	Current	Emergency	Elective	$f_{11}$	$f_{12}$	$f_{13}$
1	3	50	45	1	0.4	0.71
2	3	16	80	1	0.16	0.3
3	3	45	55	1	0.21	0.61
4	3	54	42	1	0.49	0.75
5	3	19	73	1	0.1	0.28
6	3	33	65	1	0.3	0.62
7	3	39	59	1	0.31	0.65
8	3	47	50	1	0.38	0.67
9	3	33	65	1	0.3	0.62
10	3	26	69	1	0.18	0.38
11	3	19	73	1	0.1	0.28
12	3	39	59	1	0.31	0.65
Avg.	3	35	61.25	1	0.27	0.54

**Figure 10:**
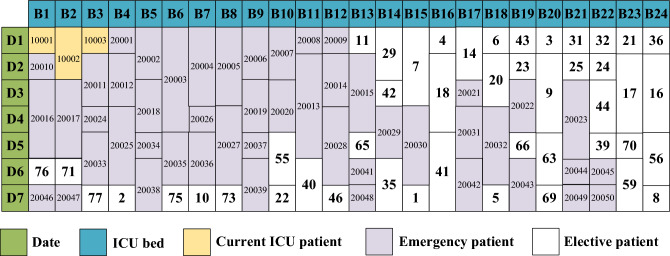
Bed allocation of solution 1.

**Figure 11:**
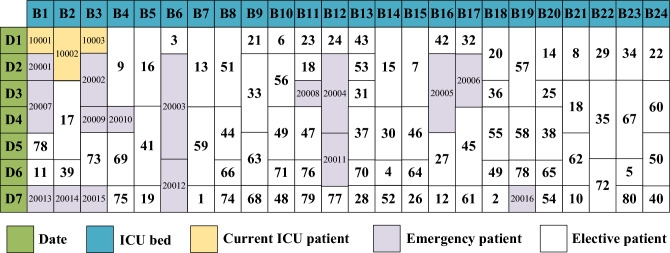
Bed allocation of solution 2.

Figure 10 depicts the bed allocation for solution 1 from Table 3. For instance, ICU bed 16 (B16) is occupied by elective patient 4 on day 1, then by elective patient 18 from days 2 to 4, and subsequently by elective patient 41 from days 5 to 7. Owing to the high emergency admission rate, a majority of the beds are taken up by emergency patients. Conversely, Fig. 11 displays solution 2 from Table 3. Here, the emergency admission rate is low, leading to most of the beds being occupied by elective patients.

**Figure 12:**
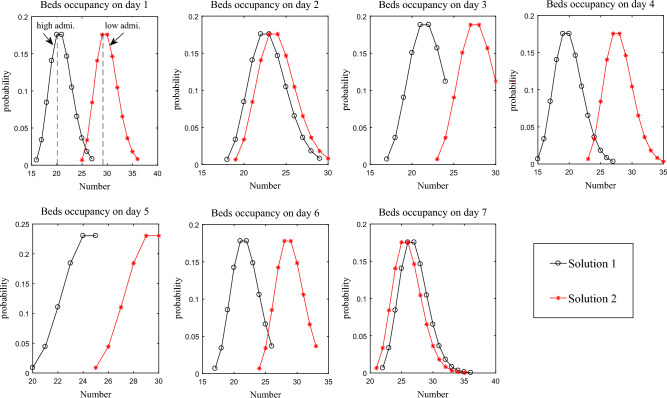
Bed occupancy of the first 2 solutions.

Figure 12 displays the probability curve of bed occupancy for the first two solutions listed in Table 3. Given the number of emergency patients is uncertain, the bed occupancy is also uncertain. For instance, on day 1, there are 3 current ICU patients and 8 elective patients. Based on the Poisson distribution, there’s a 3% probability that 1 emergency patient will arrive, and then the probability of occupying 12 beds is 3%. If the probability of 2 emergency patients arriving is 5%, then the probability of occupying 13 beds stands at 5%, and so on. The Poisson distribution indicates that while there’s low possibility for a large number of emergency patients to arrive, this could result in occupancy exceeding the available 24 beds when all emergency patients are admitted.

Under normal situation, hospitals might opt for solution 2, allocating more beds to elective patients while reserving an adequate number for emergencies. The bed occupancy remains stable from days 1–7, with the highest probability centering around occupying 28 beds. However, in critical situations, such as during the COVID-19 outbreak, hospitals could prioritize solution 1 to ensure enough beds are available for emergency patients. The probability of occupying about 20 beds is highest on days 1–4. On days 5–7, the probability of occupying about 25 beds is the highest, indicating an increase in the number of emergency patients during that period.

### Result analysis of the second stage

Some patients may extend LOS due to changes in their condition. In the second stage, we aim to extend patients’ LOS based on a probability distribution, derived from historical patient records, as shown in Table 5. Subsequently, we check whether the optimized solution in the first stage still satisfies the bed constraints. Three strategies have been designed to find robust solutions.

**Table 5:** The probability distribution of extending LOS.

Extend LOS	Probability (%)
0	40
1	25
2	20
3	10
4	5

#### Three strategies in the second stage

- Elective patient-first strategy: The needs of elective patients are prioritized over those of emergency patients. Due to this prioritization, some new emergency patients arriving later may be rejected.
- Emergency patient-first strategy: The needs of emergency patients are addressed first, followed by those of elective patients. Consequently, some elective patients might not receive admission.
- Recall algorithm to generate a new solution: Based on the patients’ extended LOS, the NSGA-II is recalled. This algorithm pursues two objectives: maximizing the similarity between the new and the first-stage solutions, and minimizing the number of added beds. It also ensures that the three objectives of the first stage do not deteriorate.

**Figure 13:**
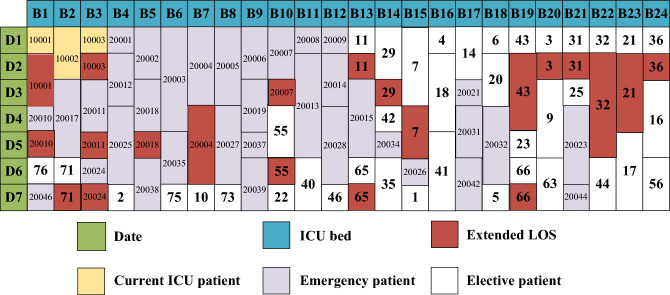
Bed allocation of solution 1 under strategy 1.

Figure 13 shows the new bed arrangement for solution 1 from Table 3 when the LOS is extended under the Elective patient-first strategy. The bed arrangements of these two solutions have an 86% similarity.

**Figure 14:**
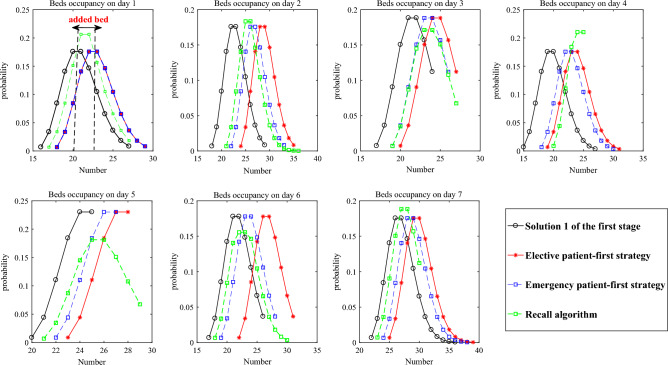
Bed occupancy of solution 1 and its three corresponding solutions.

Figure 14 compares the bed occupancy of solution 1 with its three new solutions derived from the three strategies. Given that the LOS for patients is extended, additional beds are necessary to accommodate the patients’ needs, leading the three new solutions to have a greater number of beds than solution 1.

“Elective patient-first strategy” adds the greatest number of beds, because the demands of emergency patients are not fully met and the number of new emergency patients is also uncertain. In contrast, “Recall algorithm” added the fewest beds, demonstrating its effective performance even in the second stage.

#### Results analysis of the second stage

Table 6 displays the Pareto first-front solutions from the first stage and their three corresponding solutions from the second stage. It primarily includes the numbers of admitted emergency and elective patients, as well as the two objectives of the second stage: similarity and added beds. Given that the demands of current ICU patients must be met, the number of these patients remains consistent across all solutions.

**Table 6:** The solutions and their corresponding three solutions.

No.	First-stage		Second-stage											
	Original solution		Elective patient-first				Emergency patient-first				Recall algorithm			
	Eme.	Ele.	Eme.	Ele.	$f_{21}$	$f_{22}$	Eme.	Ele.	$f_{21}$	$f_{22}$	Eme.	Ele.	$f_{21}$	$f_{22}$
1	50	45	40	43	0.86	19	45	29	0.73	15	55	26	0.38	12
2	16	80	7	60	0.77	14	12	49	0.61	11	16	55	0.36	8
3	45	55	29	40	0.88	22	38	33	0.63	14	38	42	0.36	7
4	54	42	4	57	0.87	20	50	23	0.63	15	35	40	0.49	9
5	19	73	11	60	0.75	13	19	45	0.67	13	15	52	0.47	10
6	33	65	14	58	0.82	16	28	39	0.63	14	44	37	0.33	8
7	39	59	20	46	0.88	20	35	34	0.62	18	26	50	0.52	12
8	47	50	31	40	0.84	22	44	25	0.56	17	41	35	0.38	9
9	33	65	11	60	0.82	18	28	39	0.63	12	42	43	0.33	11
10	26	69	5	62	0.80	16	26	42	0.64	15	15	57	0.46	8
11	19	73	4	57	0.75	10	19	45	0.67	11	10	70	0.47	9
12	39	59	14	58	0.88	20	35	34	0.62	16	40	57	0.56	10
**Avg.**	35	61**.**25	15.83	53.42	**0.83**	**16.79**	31.58	36.42	**0.64**	**14.25**	31.42	47.00	**0.43**	**9.01**

Two objectives of the second stage values are in bold.

**Figure 15:**
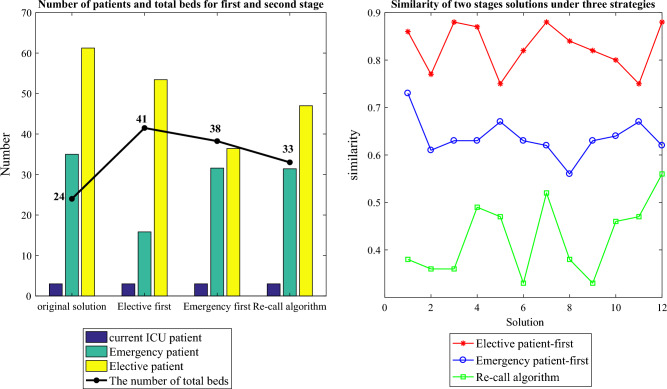
The number of patients and total beds and the similarity.

Figure 15 shows the average value of the 12 solutions about the number of admitted patients, the total ICU beds and the similarity between the original solution and the new one under the three strategies.

In Fig. 15, the three strategies require 41, 38, and 33 ICU beds, respectively, to accommodate the extended LOS of various patients and the uncertain number of emergency patients. The “Elective patient-first strategy” has a high similarity, but it requires a greater number of added beds and will reject some emergency patients. The “Emergency patient-first strategy” exhibits smaller similarities and a moderate number of added beds but may postpone more elective patients. Meanwhile, the “Recall algorithm strategy” demonstrates the least similarity and the fewest added beds, yet it offers the best admission rates for both elective and emergency patients.

## Comparison experiments

### Comparisons based on algorithms

Bed allocation performance is also influenced by the efficacy of the multi-objective algorithm. In our study, we compared NSGA-II with two other algorithms: multi-objective simulated annealing (MOSA) and multi-objective Tabu search (MOTS). To ensure a fair comparison, all three algorithms used same coding methods and optimization objectives. Additionally, domain operations in MOSA and MOTS are consistent with the crossover and mutation operations of NSGA-II.

Simulated Annealing (SA) occasionally accepts solutions that are inferior to the current one based on a certain probability. This feature can help the algorithm escape local optima and potentially find a global optimum^35,36^. The pseudocode for MOSA is detailed in Algorithm 3.

**Algorithm 3:**
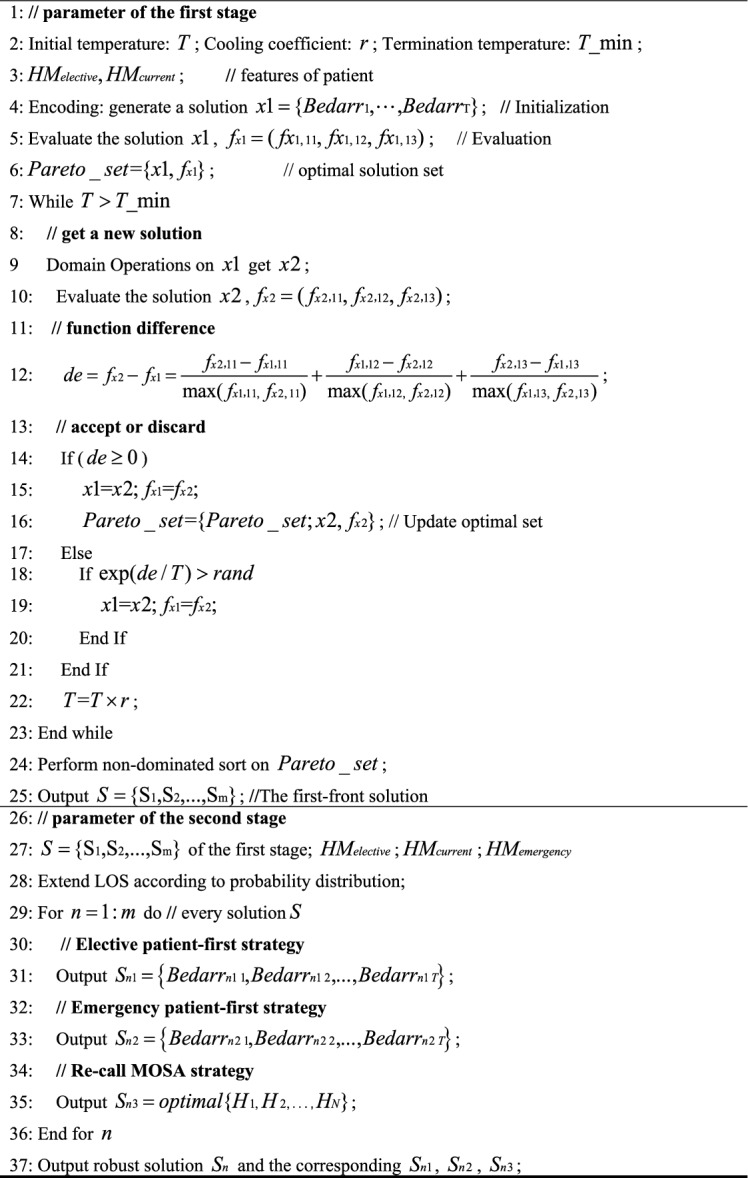
MOSA for the first and second stages.

Tabu Search (TS) is a step-by-step optimization algorithm that conducts a global neighborhood search. It emulates human memory by marking previously identified local optimal solutions and processes during the search. By doing so, it avoids revisiting them in subsequent searches^37,38^. The pseudocode for MOTS is detailed in Algorithm 4. The parameters of MOSA and MOTS algorithms are set according to the literatures^39,40^, as shown in Table 7.

**Table 7:** Parameters of MOSA and MOTS.

	Parameters	Value
MOSA	Start temperature	1000
	End temperature	1.0e−3
	Cooling rate	0.95
MOTS	Tabu list	50
	Tabu length	5
	Candidate set	10
	Iterations	200

**Algorithm 4:**
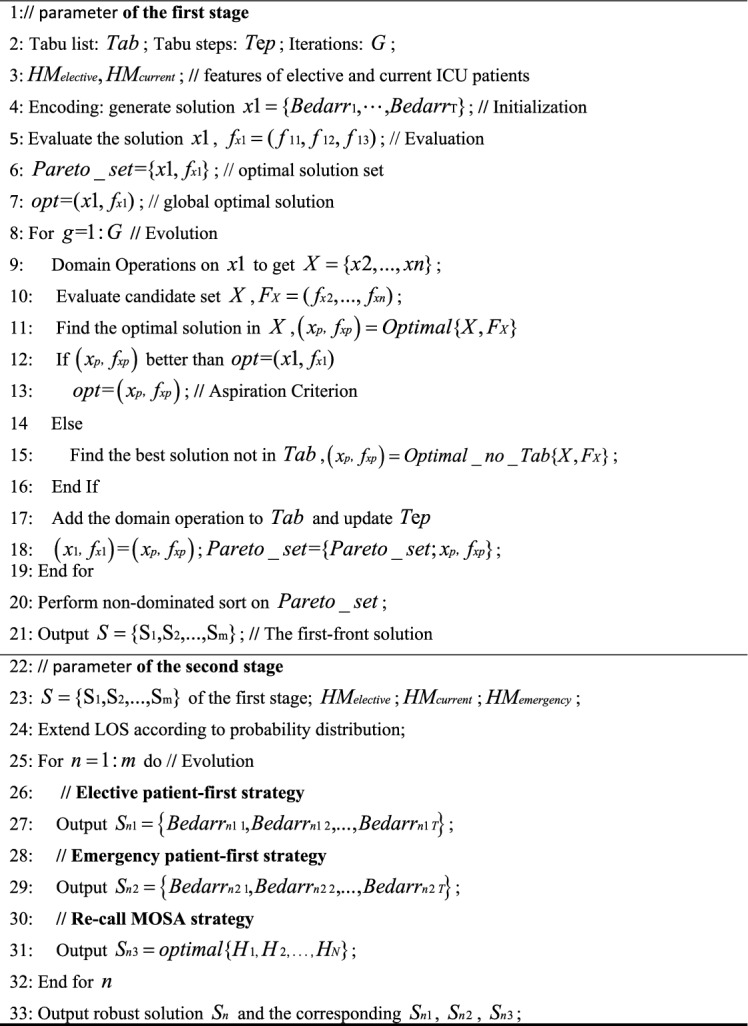
MOTS for the first and second stages.

#### Result analysis

**Figure 16:**
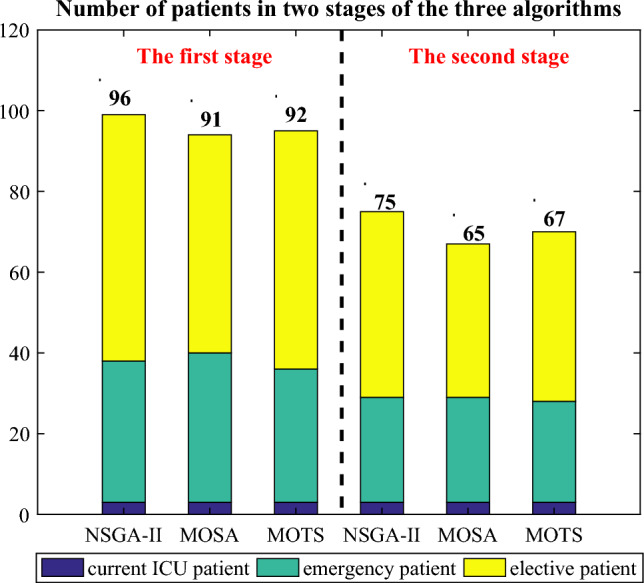
The average number of patients in all solutions of the three algorithms.

NSGA-II, MOSA, and MOTS are implemented in Matlab R2014a on Windows 10 (X64). The best result is chosen from 10 runs. Figure 16 displays the average number of the three patient types across all solutions using the three algorithms. Due to some patients extending their LOS in the second stage, the number of admitted patients in this stage is fewer than in the first stage. NSGA-II outperforms both MOSA and MOTS, resulting in more patients being admitted to the ICU.

Table 8 presents the three optimization objectives from the first stage and the two optimization objectives from the second stage for the MOSA and MOTS algorithms. In the second stage, the averages for “Similarity” and “Added bed” are computed across the three strategies. The “Added beds” are 13.35, 16.93, and 14.78, indicating that, on average, NSGA-II, MOSA, and MOTS require 37, 41, and 39 beds respectively to satisfy patient demands.

**Table 8:** The optimization objectives in the two stages of MOSA and MOTS.

No.	MOSA					MOTS				
	The first stage			The second stage		The first stage			The second stage	
	$f_{11}$	$f_{12}$	$f_{13}$	$f_{21}$	$f_{22}$	$f_{11}$	$f_{12}$	$f_{13}$	$f_{21}$	$f_{22}$
1	1	0.22	0.38	0.46	14.81	1.00	0.39	0.39	0.82	11.98
2	0.98	0.2	0.27	0.62	18.25	0.98	0.28	0.38	0.33	16.53
3	1	0.74	0.54	0.69	16.02	1.00	0.69	0.49	0.57	14.47
4	1	0.81	0.55	0.56	17.65	0.98	0.27	0.36	0.56	13.88
5	1	0.23	0.5	0.47	16.5	1.00	0.43	0.42	0.73	16.52
6	1	0.46	0.51	0.58	18.54	0.99	0.47	0.45	0.5	15.89
7	1	1.25	0.61	0.58	19.85	1.00	0.65	0.46	0.65	13.04
8	0.99	0.29	0.51	0.76	16.97	1.00	0.71	0.52	0.7	16.64
9	1	0.65	0.53	0.57	15.12	1.00	0.7	0.5	0.67	14.02
10	1	0.82	0.58	0.61	17.08	1.00	0.75	0.64	0.63	13.88
11	0.99	0.32	0.52	0.43	16.62	1.00	0.8	0.66	0.58	15.4
12	1	0.65	0.53	0.61	15.69	1.00	0.28	0.36	0.54	15.74
Avg.	1	0.55	0.50	0.58	16.93	1	0.51	0.47	0.61	14.78

**Figure 17:**
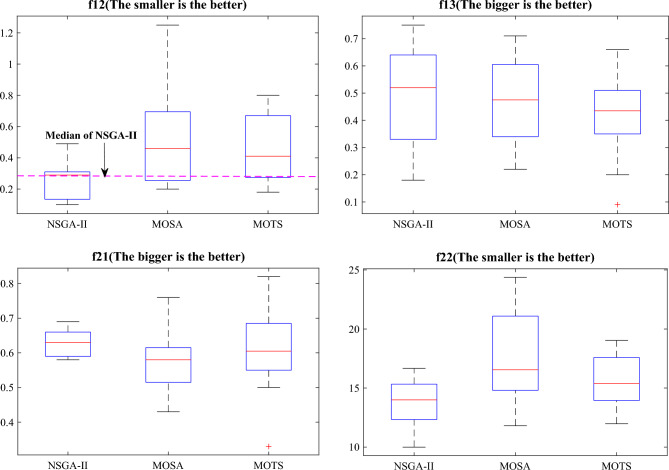
The boxplots of the four optimization objectives.

Figure 17 shows the boxplots of the four optimization objectives except $f_{11}$ for the three algorithms. The median of MOSA and MOTS is different from the median of NSGA-II, and NSGA-II can explore more possibilities and better solutions. The average running time of the three algorithms is 41.42 s, 24.65 s, and 32.74 s, respectively. NSGA-II has the best results and the longest run time, but this time is acceptable.

#### Statistical analysis

In addition, a paired t-test was conducted to determine whether there are any statistically significant differences between NSGA-II and the two other algorithms: MOSA and MOTS. Given the expectation that the solution quality of NSGA-II is superior to that of the other two algorithms, a one-sided alternative hypothesis H1 was formulated as follows,

$${\text{H}}1{:}\;\;\mu_{NSGA{\text{-}}II} - \mu_{OA} < 0$$

where $\mu_{NSGA{\text{-}}II}$ and $\mu_{OA}$ are the population means for NSGA-II and OA (representing the compared algorithms: MOSA and MOTS). The results of the paired t-test are listed in Table 9.

**Table 9:** Results of paired t-test.

Problem	NSGA-II VS MOSA	NSGA-II VS MOTS
Mean difference	− 3.58	− 1.43
P value	0.012	0.036

As shown in Table 9, for all three pairs, P was less than 0.1, and the mean difference is less than 0, which indicates that NSGA-II is significantly different from other algorithms and performs better.

### Comparisons based on scenarios

#### Scenario design

Probabilistic distributions of extending LOS have some implications for bed allocation in the second stage. Table 10 shows the probability distribution of extending LOS through 5 representative scenarios. For example, S1 represents the situation in which “Extend LOS = 0 day” accounts for the largest proportion, and S2 represents the situation in which “Extend LOS = 1 day” accounts for the largest proportion, etc. The last row in Table 10 is the probability distribution curve of the scenarios with five trends respectively.

**Table 10:** The probability distributions under 5 scenarios.

Extend LOS	S1	S2	S3	S4	S5
0	40%	25%	5%	5%	5%
1	25%	40%	25%	10%	10%
2	20%	20%	40%	20%	20%
3	10%	10%	20%	40%	25%
4	5%	5%	10%	25%	40%
Trend					

#### Result analysis

**Figure 18:**
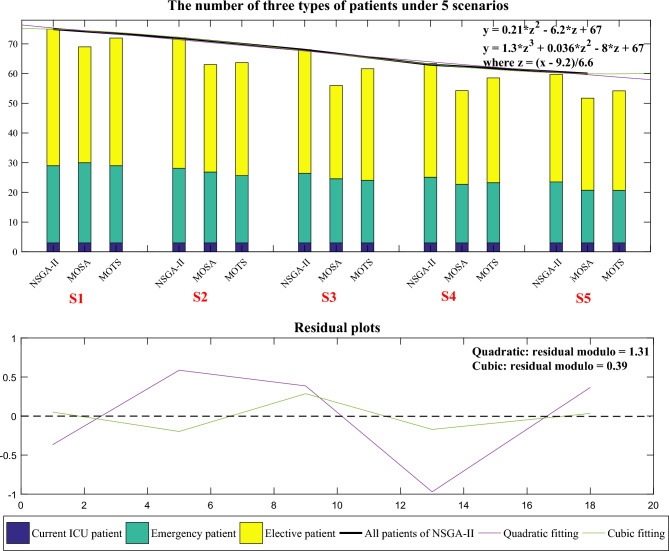
The number of admitted patients of the three algorithms under 5 scenarios.

**Figure 19:**
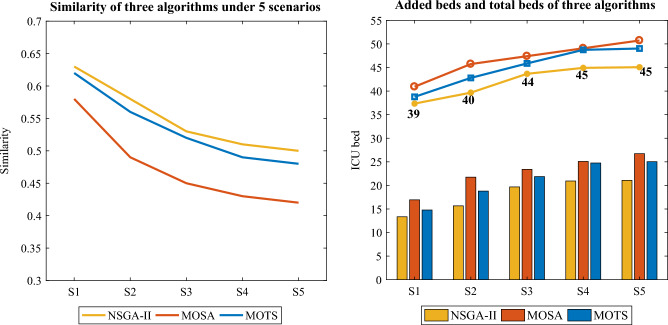
Similarity and added and total beds of the three algorithms under 5 scenarios.

Figure 18 shows the total number of admitted patients of the three algorithms under the 5 scenarios. Compared with MOSA and MOTS, the changes of NSGA-II are gentler, indicating that NSGA-II is fit to generate robust solutions. To analyze the variations in patient numbers in the 5 scenarios, the number of admitted patients using NSGA-II were fitted. Both quadratic and cubic fits were found to be the better fits. Figure 19 presents the fitted curve and equation, with the residual plot revealing that the modulus of the residuals for the cubic fit is a mere 0.39. NSGA-II can fit the number of admitted patients in different scenarios, which shows that when the scenario changes appropriately, NSGA-II can still obtain reasonable results, which indicates that when solving ICU bed allocation under uncertainty, our model and algorithm are robust.

Table 11 shows the results of the five optimization objectives in the two stages using the three algorithms under the 5 scenarios, and Fig. 19 shows the similarity and added beds for the three algorithms. In scenario 5, where most patients extend their LOS, there’s a significant shift in bed allocation, leading to a lower similarity and a greater need for additional beds. Conversely, in scenario 1, with fewer patients extending their LOS, bed allocation changes minimally, resulting in higher similarity and fewer additional beds. The “added beds” of NSGA-II are 13.35, 15.67, 19.67, 20.93, and 21.07, respectively, indicating that in 5 scenarios, to meet the demands of patients, 37, 40, 44, 45, 45 ICU beds are required respectively.

**Table 11:** Five objectives of the three algorithms under 5 scenarios.

No.	Stage	Obj.	Scenario				
			S1	S2	S3	S4	S5
NSGA-II	The first stage	$f_{11}$	1	1	1	1	1
		$f_{12}$	0.27	0.27	0.27	0.27	0.27
		$f_{13}$	0.54	0.54	0.54	0.54	0.54
	The second stage	$f_{21}$	0.63	0.58	0.53	0.51	0.50
		$f_{22}$	13.35	15.67	19.67	20.93	21.07
MOSA	The first stage	$f_{11}$	1	0.99	0.99	0.99	0.99
		$f_{12}$	0.55	0.55	0.55	0.55	0.55
		$f_{13}$	0.5	0.5	0.5	0.5	0.5
	The second stage	$f_{21}$	0.58	0.49	0.45	0.43	0.42
		$f_{22}$	16.93	21.74	23.41	25.08	26.73
MOTS	The first stage	$f_{11}$	1	0.99	0.99	0.99	0.99
		$f_{12}$	0.51	0.51	0.51	0.51	0.51
		$f_{13}$	0.47	0.47	0.47	0.47	0.47
	The second stage	$f_{21}$	0.61	0.57	0.52	0.49	0.48
		$f_{22}$	14.78	18.8	21.87	24.75	25.03

**Figure 20:**
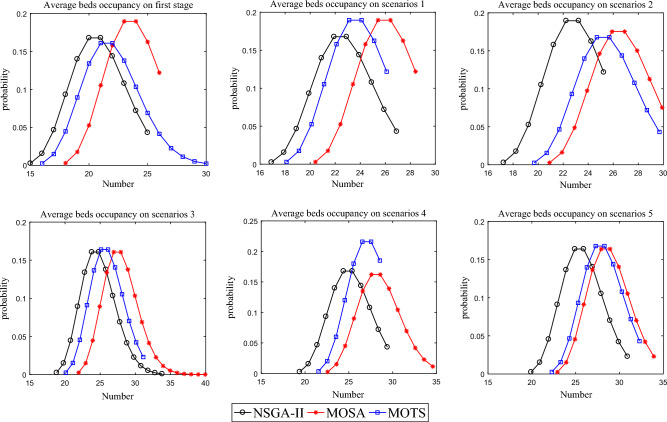
The average bed occupation of the three algorithms.

Figure 20 shows the average bed occupation using three algorithms in the first stage and the 5 scenarios in the second stage. The bed occupation curve of NSGA-II lies to the left of the curve for MOSA and MOTS, indicating that NSGA-II requires fewer ICU beds compared to MOSA and MOTS.

## Conclusion

This paper studies ICU bed arrangement when the number of emergency patients is uncertain and the LOS of three types of patients is also uncertain. The research is conducted in two primary stages. The first stage considers how to balance reducing elective patient waiting time while increasing emergency admission rate and bed utilization. Based on the first stage, the second stage considers how to minimize the number of added beds while ensuring the least changes in bed arrangements when patients extend their LOS.

This research improves the utilization rate of medical resources and the stability of hospital scheduling. This can lead to decreased hospital expenses, the accommodation of more patients, and heightened patient satisfaction. Moreover, this research provides strategies for hospitals to effectively respond to varying demands for ICU beds during sudden illnesses. A limitation of this research is its assumption that emergency patient arrivals follow a Poisson distribution. Future studies should explore fitting other distributions for emergency patient arrivals.

## Data Availability

Datasets used and/or analyzed during this study are available upon reasonable request to the corresponding author.
